# Impact of Storage Condition on Chemical Composition and Antifungal Activity of Pomelo Extract against *Colletotrichum gloeosporioides* and Anthracnose in Post-harvest Mango

**DOI:** 10.3390/plants11152064

**Published:** 2022-08-07

**Authors:** Yu-Jung Cheng, Ying-Jou Wu, Fang-Wei Lee, Ling-Yi Ou, Chi-Nan Chen, Yu-Ying Chu, Yen-Chou Kuan

**Affiliations:** 1 Department of Horticulture and Landscape Architecture, National Taiwan University, Taipei 10617, Taiwan; 2 Chiayi Agricultural Experiment Branch, Taiwan Agricultural Research Institute, Council of Agriculture, Executive Yuan, Chiayi 60044, Taiwan; 3 Highland Experimental Farm, National Taiwan University, Nantou 54641, Taiwan

**Keywords:** anthracnose, antifungal, edible coating, mango, pomelo peel, post-harvest disease

## Abstract

Anthracnose caused by *Colletotrichum* leads to a tremendous post-harvest mango loss. While chemical fungicides are applied to control anthracnose, natural alternatives are preferred due to food safety and environmental concerns. Pomelo extract (PE) exhibits a broad spectrum of antimicrobial activities; however, its effect against anthracnose is unknown. Here we investigated the chemical profile of PE using GC-MS and the anti-anthracnose activity of PE using in vitro and in vivo assays. We also evaluated the impact of storage temperature (0°, 5°, 10°, 20°, −20°, and −80 °C) and light conditions on the composition and antifungal activity of PE. We found that PE inhibited *C. gloeosporioides* in vitro with an IC_50_ of 3.2 mL L^−1^. Applying chitosan-based coating incorporated with 20 mL L^−1^ PE significantly suppressed anthracnose in post-harvest ‘Keitt’ mango. A storage temperature below 5 °C substantially preserved major compounds and the antifungal activity of PE after 6 m of storage. Finally, we showed that applying *d*-limonene, the key constituent of PE, inhibited *C. gloeosporioides* in vitro (IC_50_: 10.9 mM) and suppressed anthracnose in vivo. In conclusion, we demonstrated that the application of PE and *d*-limonene are sustainable methods for anthracnose control in post-harvest crops and established the preservation protocol for PE.

## 1. Introduction

Pomelo (*Citrus grandis* Osbeck), also known as pummelo or shaddock, is a fruit crop belonging to the *Rutaceae* family. Pomelo is cultivated and consumed worldwide as fresh fruit or processed products such as juice and cans [1]. Pomelo fruit has a characteristic of thick peel, which may account for over 30% of fresh weight. Consequently, the consumption of pomelo generates a large amount of agricultural waste. To cope with this problem, several pomelo by-products have been developed, including essential oil, pectin, food additives, phytochemicals, and absorbents [2].

Mango (*Mangifera indica* L.) is a tropical fruit appreciated worldwide for its high nutrition value and pleasant sensory qualities. The export of fresh mango fruit from the production regions such as Southeast Asia and South America to the markets in the temperate regions requires long-distance shipments. During the period of transportation and marketing, mango fruit suffers from severe loss caused by anthracnose, which is the most common fungal disease in post-harvest mango [3]. The disease is caused by infection of *Colletotrichum* species to the immature fruit. The pathogen remains quiescent until the fruit begins to ripen, causing disease symptoms such as the development of lesions and sunken fruit skin, severely damaging the quality and value of the fruit [4].

Fungicides such as Prochloraz^®^ are applied to fruit crops such as mango, papaya, and avocado, which are susceptible to post-harvest anthracnose decay [5,6,7]. However, due to environmental and food safety considerations, alternative methods using non-synthetic or materials extracted from natural sources for anthracnose control have been explored. These include the application of nanoparticles [8,9] chitosan-based coatings [10], and essential oil formulations [3].

The antifungal effect of essential oil against *C. gloeosporioides* has been demonstrated in several in vitro and in vivo studies. Essential oil of rosemary, noni, and savory plant inhibited the growth of *C. gloeosporioides* isolated from papaya [11,12]. Lemongrass oil vapor reduced anthracnose decay of papaya [13]. Thyme oil inhibited the growth of *C. gloeosporioides* isolated from mango [14] and suppressed anthracnose in mango [15]. Chitosan-thyme oil nanoparticles exerted synergistic effects on inhibiting *C. gloeosporioides* growth in vitro and suppressed anthracnose in avocado [16].

The antimicrobial activities of essential oil isolated from pomelo and other citrus fruits have also been reported. In in vitro studies, pomelo essential oil inhibited *Staphylococcus aureus* and *Escherichia coli* growth [17], while grapefruit essential oil inhibited *E. coli*, *S. aureus*, *Bacillus subtilis*, and *Salmonella typhimurium* growth [18]. In an in vivo study, mandarin (*Citrus reticulata*) essential oil exhibited antifungal activity against *Aspergillus niger* in onion bulbs [19].

Despite extensive research on the application of essential oil as natural antimicrobial agents against post-harvest anthracnose, the antifungal activity of pomelo essential oil on *C. gloeosporioides* remains unclear. Here, we examined the composition of pomelo peel extract and evaluated its antifungal effect against *C. gloeosporioides* isolated from mango and in post-harvest mango fruit. We also investigated the effect of storage conditions on the composition of pomelo extract. In addition, we demonstrated that *d*-limonene was the active compound and confirmed its antifungal activities through in vitro and in vivo experiments.

## 2. Results

### 2.1. Chemical Composition of Pomelo Extract

**Figure 1:**
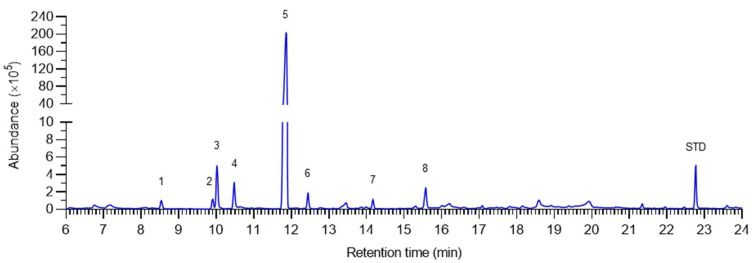
Chromatograph showing the chemical profile of pomelo extract. The numbers indicate the compounds described in Table 1, and STD indicates the internal standard ethyl decanoate.

The chemical composition of the freshly prepared PE sample was determined by GC-MS analysis. The chromatograph showed 8 major peaks (Figure 1), and the identity of each peak was determined by comparing the retention time and the fragmentation pattern with the NIST database, and the retention indices of the identified compounds were calculated (Table 1). The abundance of each constituent was calculated based on the area of the signal and expressed as a percent of the total in Table 1. The main compounds were *α*-pinene (0.37%), sabinene (0.37%), *β*-pinene (1.8%), *β*-myrcene (0.91%), *d*-limonene (76.1%), *β*-ocimene (0.51%), linalool (0.31%), and 2,3-Dihydro-3,5-dihydroxy-6-methyl-4H-pyran-4-one (0.78%). The concentrations of the major compounds were calculated by normalization with 100 μL L^−1^ ethyl decanoate internal control (Table 1). The concentration of *d*-limonene, the dominant constituent of PE, was 7446 μL L^−1^ (46.5 mM).

### 2.2. Pomelo Extract Inhibits C. gloeosporioides In Vitro and in ‘Keitt’ Mango

**Figure 2:**
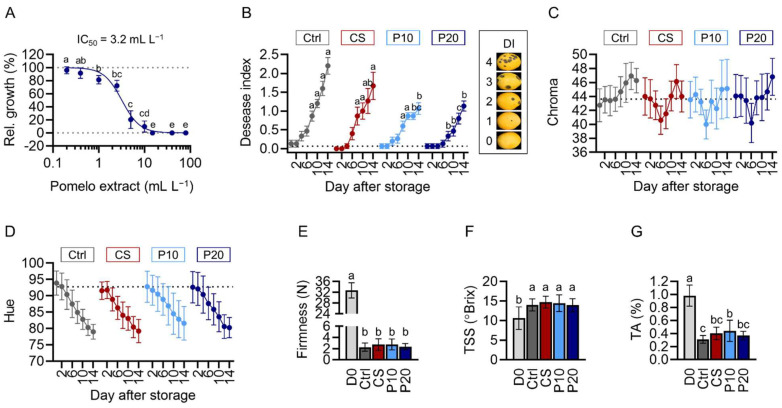
Pomelo extract inhibits *Colletotrichum gloeosporioides* in vitro and in vivo. (**A**) The inhibition curve and IC_50_ of PE against *C. gloeosporioides* determined by in vitro assays. (**B**) The disease index of fruit treated with water, chitosan solution (CS), CS incorporated with 10 mL L^−1^ PE (P10), or 20 mL L^−1^ PE (P20). (**C**–**G**) The physiological traits of fruit. The chroma (**C**) and hue (**D**) of the fruit surface determined by a CR-400 chromameter. The firmness (**E**) of fruit determined by a TA.XTplus Texture Analyser. The total soluble solids (**F**) and titratable acids (**G**) determined by a PAL-1 digital refractometer and a Titrando automated titration system, respectively. Data are presented as mean ± SD (**A**,**C**–**G**) or SEM (**B**). Different alphabets indicate significant difference between data (Tukey’s tests, *p* < 0.05; (**A**): *n* = 5; (**B**–**G**): *n* = 15).

Essential oil isolated from pomelo and citrus fruits exhibited antifungal activates against a wide range of food-borne or plant pathogens. However, the effect of pomelo peel components on *C. gloeosporioides* is unclear. To investigate the antifungal effect of PE, we prepared PDA plates incorporated with 0–80 mL L^−1^ PE and inoculated *C. gloeosporioides* onto the plates to monitor fungal growth. As demonstrated by the inhibition curve, PE displayed significant inhibitory activity at 1 mL L^−1^ and completely inhibited fungal growth at over 20 mL L^−1^. The estimated IC_50_ of PE was 3.2 mL L^−1^ (Figure 2A).

To confirm the anti-anthracnose efficacy, mango fruit was treated with water, CS, or CS incorporated with 10 mL L^−1^ PE (P10) or 20 mL L^−1^ PE (P20). Disease symptoms appeared after 4 d in water-treated, CS-treated, and P10-treated fruit, whereas disease symptoms appeared after 8 d in P20-treated fruit (Figure 2B). The water-treated and CS-treated fruit displayed similar disease severities throughout the experiment, whereas the P20-treated fruit showed significantly alleviated disease severities after 8 d of storage (Figure 2B). The disease severity of P10-treated fruit was also substantially alleviated after 12 d and 14 d of storage compared to water- and CS-treated fruit (Figure 2B).

The surface color, firmness, total soluble solids, and titratable acids were also analyzed to examine the effects of coating treatments on fruit ripening during storage. The chroma of water-treated fruit increased steadily during storage. Notably, all the coating treatments altered the chroma pattern, in which the chroma decreased during the first 6 d and then increased after 6 d of storage (Figure 2C). After 14 d of storage, the hue of the fruit surface decreased from 92.8 ± 4.1° to 77 ± 3.7° (Figure 2D) and the fruit firmness reduced from 32.7 ± 2.8 N to 2.5 ± 0.9 N (Figure 2E), whereas the total soluble solids increased from 10.6 ± 2.9° Brix to 14.3 ± 1.7° Brix (Figure 2F), and the titratable acids diminished from 1.0 ± 0.2% to 0.4 ± 0.1% (Figure 2G). No statistical difference was observed in hue, firmness, total soluble solids, or titratable acids between water-treated and coating-treated fruit, indicating that the coating treatments did not alter the ripening of the mango fruit during storage.

In addition, we tested the antifungal effect of ethanol through in vitro and in vivo experiments. In the in vitro assay, ethanol inhibited *C. gloeosporioides* growth at 5 mL L^−1^ and completely inhibited fungal growth at 80 mL L^−1^ with an estimated IC_50_ of 17.5 mL L^−1^. (Supplementary Figure S1). However, applying CS incorporated with 20 mL L^−1^ ethanol on mango displayed no inhibitory effect on anthracnose development but induced pitting and skin darkening of the fruit 2 d after storage (Supplementary Figure S2).

### 2.3. Change in Main Constituents of Pomelo Extract Stored under Different Conditions

**Figure 3:**
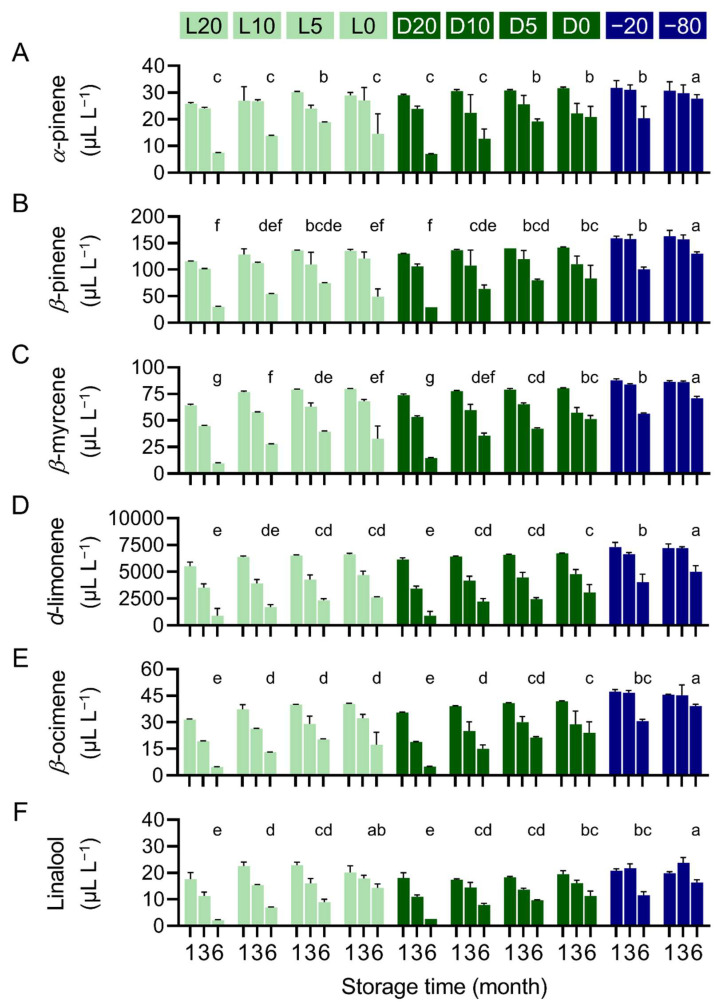
Change in main constituents of pomelo extract stored under different conditions. (**A**) *α*-pinene, (**B**) *β*-pinene, (**C**) *β*-myrcene, (**D**) *d*-limonene, (**E**) *β*-ocimene, and (**F**) linalool concentration in the PE samples stored under indicated conditions were determined by GC-MS. Data are presented as mean ± SD. The difference between values of 6 m storage was analyzed by Tukey’s tests. Significant differences (*p* < 0.05, *n* = 3) are indicated by different alphabets.

The concentration of *α*-pinene in the PE samples held in 20° and 0 °C storage rooms reduced to 71.1% and 79.6% of the initial concentration after 1 m and dropped to 20.9% and 40.2% after 6 m, respectively. When stored in the dark, *α*-pinene concentration decreased to 80.0% and 87.3% after 1 m and to 19.5% and 57.6% after 6 m, respectively. When kept in −20° and −80 °C freezers, *α*-pinene concentration was maintained at 85.9% and 82.1% after 3 m, and decreased to 56.5% and 76.6% after 6 m, respectively (Figure 3A).

The concentration of *β*-pinene in the PE sample dropped to 66.0% and 77.8% of the initial value after 1 m and gradually diminished to 17.2% and 46.2% after 6 m at 20 °C and 5 °C, respectively. When protected from light, *β*-pinene concentration dropped to 74.4% and 80.8% after 1 m and diminished to 16.8% and 47.5% after 6 m at 20 °C and 0 °C, respectively. When stored at −20° and −80 °C, *β*-pinene concentration was maintained at 90.1% and 89.5% after 3 m, and reduced to 57.6% and 74.2% after 6 m, respectively (Figure 3B).

The concentration of *β*-myrcene in the PE samples stored at 20° and 0 °C decreased to 68.0% and 84.4% after 1 m, and diminished to 10.3% and 41.8% after 6 m, respectively. When protected from light, *β*-myrcene concentration reduced to 78.2% and 85.0% after 1 m and dropped to 15.6% and 54.4% after 6 m storage at 20° and 0 °C, respectively. When held in −20° and −80 °C freezers, *β*-myrcene concentration was maintained at 88.6% and 91.1% after 3 m, and decreased to 59.8% and 74.9% after 6 m, respectively (Figure 3C).

The concentration of *d*-limonene decreased to 74.8% of the initial value after 1 m, and dropped to 12.3% after 6 m in the PE samples stored at 20 °C. When stored at 0 °C, *d*-limonene concertation slightly reduced to 90.0% after 1 m and then steadily decreased to 35.7%. Protection from light displayed a minor effect on the maintenance of *d*-limonene, whose concentration dropped to 12.2% and 41.8% after 6 m storage in the dark at 20 and 0 °C, respectively. PE samples stored at −20° and −80 °C maintained *d*-limonene concentration at 90.2% and 97.8% after 3 m storage, respectively. The *d*-limonene concentration was 55.0% and 67.6% for the samples held at −20° and −80 °C for 6 m, respectively (Figure 3D).

The concentration of *β*-ocimene in the PE samples stored at 20° and 0 °C reduced to 64.0% and 82.2% of the initial value after 1 m, and to 9.7% and 35.3% after 6 m, respectively. When protected from light, the *β*-ocimene concentration decreased to 72.3% and 85.2% after 1 m, and to 10.1% and 49.0% after 6 m at 20° and 0 °C, respectively. When stored at −20° and −80 °C, *β*-ocimene concentration was maintained at 94.5% and 92.4% after 3 m, and decreased to 62.5% and 79.8% after 6 m, respectively (Figure 3E).

The concentration of linalool in the PE samples stored at 20° and 0 °C diminished to 58.4% and 66.9% of the initial value after 1 m, and to 7.3% and 47.6% after 6 m, respectively. When protected from light, linalool concentration decreased to 59.9% and 64.7% after 1 m, and to 9.0% and 37.4% after 6 m at 20 and 0 °C, respectively. When stored at −20° and −80 °C, linalool concentration reduced to 71.9% and 78.9% after 1 m, and diminished to 38.4% and 54.2% after 6 m, respectively (Figure 3F).

### 2.4. Refrigeration and Freeze Storage Retain the Main Constituents in Pomelo Extract

After 6 m storage, the concentration of all the major constituents in the PE samples stored at −80 °C was significantly higher than those held under other conditions (Figure 3). When protected from light, the PE samples kept at −20 °C showed significantly higher *d*-limonene concentration than those stored at non-freezing temperature (Figure 3D). In the case of *α*-pinene, *β*-pinene, and *β*-ocimene, the concentration was similar among the PE samples stored at 5°, 0°, and −20 °C (Figure 3A,B,E), whereas in the case of *β*-myrcene, the percent retention was similar among the PE samples stored at 0° and −20 °C (Figure 3C). In the case of linalool, the concentration was similar among the PE samples stored at 10°, 5°, 0°, and −20 °C, while storage at −80 °C retained significantly higher *β*-ocimene than all other conditions except for storage at 0 °C without light protection (Figure 3F). The effect of light protection was observed in PE samples stored at 0 °C, where protection from light led to a significantly higher retention of *α*-pinene, *β*-pinene, *β*-myrcene, and *β*-ocimene (Figure 3A–C,E). However, such an effect was not observed in *d*-limonene and linalool (Figure 3D,F).

**Figure 4:**
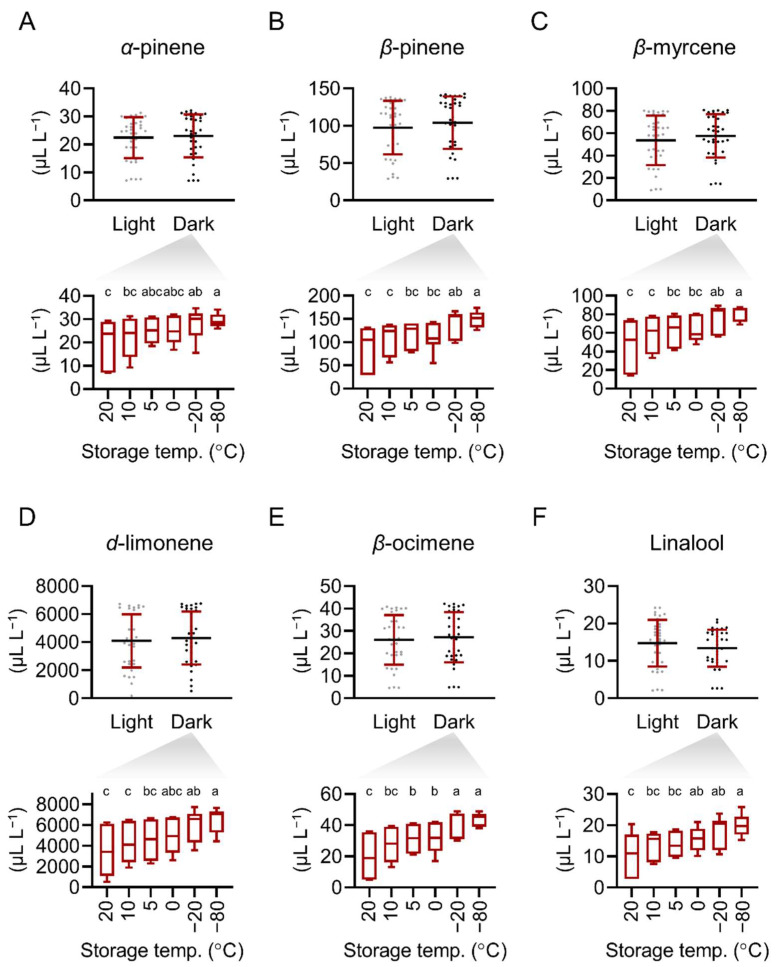
Storage light and temperature conditions affect the retention of (**A**) *α*-pinene, (**B**) *β*-pinene, (**C**) *β*-myrcene, (**D**) *d*-limonene, (**E**) *β*-ocimene, and (**F**) linalool in PE. Top panels: data are presented as mean ± SD with individual values presented in dots. Bottom panels: box and whisker plots showing minimum, first quartile, median, third quartile, and maximum values of the data. Significant differences are indicated by different alphabets (Tukey’s tests, *p* < 0.05; top panels: *n* = 36; bottom panels: *n* = 9).

To further analyze the effect of light on the major constituents of PE samples, the data of *α*-pinene, *β*-pinene, *β*-myrcene, *d*-limonene, *β*-ocimene, and linalool concentration in all the PE samples stored at 0–20 °C without light protection were pooled, and compared with the data of the PE samples stored at 0–20 °C and protected from light. We found the effect of light protection was indiscernible in all the examined constituents (Figure 4, top panels). This result demonstrates that under our experimental settings, light was a minor factor in maintaining the major constituents in PE. We next examined the effect of temperature. The concentration of the major constituents in all the samples stored with light protection were pooled for each temperature (Figure 4, bottom panels).

Decreasing storage temperature from 20° to −20° or −80 °C significantly increased the retention of all major constituents (Figure 4, bottom panels). Reducing storage temperature from 20° to 5° or 0 °C increased the retention of *β*-ocimene (Figure 4E), whereas reducing storage temperature from 20° to 0 °C increased the retention of linalool (Figure 4F). In the cases of *α*-pinene, *β*-pinene, *β*-myrcene, *d*-limonene, and linalool, storage at 5 °C improved the retention of these compounds as compared to 20 °C and 10 °C storage, and the concentrations of the constituents were similar to those at 0° and −20 °C storage (Figure 4A–D,F).

### 2.5. Antifungal Activity of Pomelo Extract Correlates to the Abundance of Main Constituents

To evaluate the effect of storage conditions on the maintenance of PE antifungal activity, PE samples stored under different conditions were sampled 1, 3, and 6 months after storage and prepared into PDA plates at the concentration of 4 mL L^−1^ for fungicidal assays.

**Figure 5:**
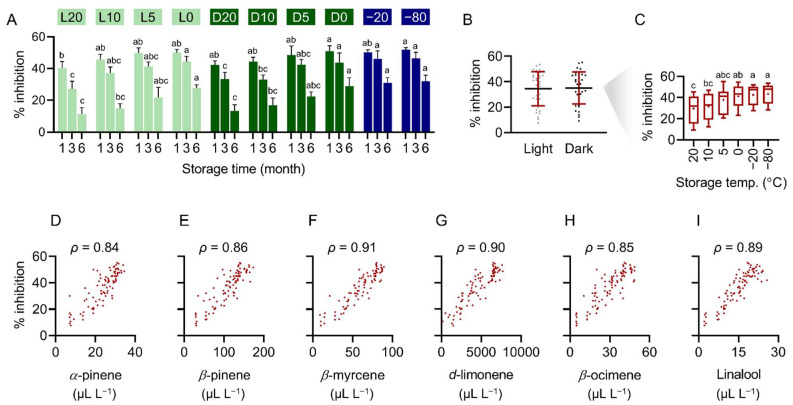
Correlation between the major constituents and antifungal activity of pomelo extract. (**A**) The relative growth of *C. gloeosporioides* on PDA plates prepared with 4 mL L^−1^ PE stored under indicated conditions and storage time. Data are presented as mean ± SD. (**B**) Antifungal activity of PE samples stored with or without light protection. Data are presented as mean ± SD and individual values as dots. (**C**) Antifungal activity of PE samples stored at 20°, 10°, 5°, 0°, −20°, and −80 °C. Data are presented in box and whisker plots showing the minimum, first quartile, median, third quartile, and maximum values, and the means of data are indicated by dots. Analysis of correlation between the antifungal activity and the concentration of (**D**) *α*-pinene, (**E**) *β*-pinene, (**F**) *β*-myrcene, (**G**) *d*-limonene, (**H**) *β*-ocimene, and (**I**) linalool. Significant differences among storage conditions are indicated by different alphabets (Tukey’s tests; *p* < 0.05, (**A**): *n* = 3; (**B**): *n* = 36; (**C**): *n* = 9; (**D**–**I**): *n* = 90).

After 1 m storage, the antifungal activity of PE samples stored at 20 °C without light protection was 40.4%, significantly lower than the average inhibitory activity of the PE samples stored under other conditions, which was 48.2% (Figure 5A). After 3 m storage, the antifungal activity of the PE samples stored without light protection at 20°, 10°, 5°, 0°, and −20 °C and with light protection at 20°, 10°, 5°, 0°, −20°, and −80 °C was 27.1%, 37.4%, 41.4%, 44.5%, 33.4%, 33.1%, 42.5%, 43.8%, 46.3%, and 46.4%, respectively (Figure 5A). After 6 m storage, the antifungal activity of PE samples stored without light protection at 20°, 10°, 5°, 0°, −20 °C and with light protection at 20°, 10°, 5°, 0°, −20°, and −80 °C was 11.3%, 15.0%, 21.9%, 27.8%, 13.3%, 16.8%, 22.4%, 29.0%, 31.0%, and 32.1%, respectively (Figure 5A).

Protection from light had a minor effect on maintaining the antifungal activity of the PE samples (Figure 5A,B), whereas decreasing temperature from 20 °C to 0 °C significantly increased the antifungal activity of the post-storage PE samples (Figure 5A,C). Notably, although we observed a clear upward trend in the antifungal activity of PE samples stored at 5°, 0°, −20°, and −80 °C, the difference between 5 °C and 0 °C, the differences between 0°, −20°, and −80 °C were nonsignificant (Figure 5A,C).

We also found strong correlations between the antifungal activity and the concentration of all the examined constituents. The correlation coefficient between the antifungal activity and the concentration of *α*-pinene, *β*-pinene, *β*-myrcene, *d*-limonene, *β*-ocimene, and linalool was 0.84, 0.86, 0.91, 0.90, 0.85, and 0.89, respectively (Figure 5D–I).

### 2.6. D-Limonene Acts as the Key Component against Mango Anthracnose

**Figure 6:**
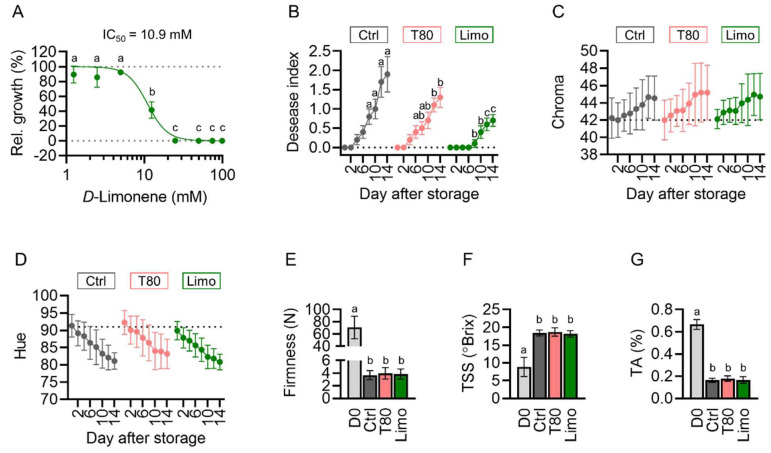
*D*-limonene inhibits *Colletotrichum gloeosporioides* in vitro and in vivo. (**A**) The inhibition curve and IC_50_ of *d*-limonene against *C. gloeosporioides* determined by in vitro assay. (**B**) The disease index of fruit treated with water, Tween^®^80 (T80), or 6.2 mM *d*-limonene (Limo). (**C**–**G**) The fruit physiology. The chroma (**C**) and hue (**D**) of the fruit surface determined by a CR-400 chromameter. The firmness (**E**) of fruit determined by a TA.XTplus Texture Analyser. The total soluble solids (**F**) and titratable acids (**G**) determined by a PAL-1 digital refractometer and a Titrando automated titration system, respectively. Data are presented as mean ± SD (**A**,**C**–**G**) or SEM (**B**). Significant differences are indicated by different alphabets (Tukey’s tests, *p* < 0.05; (**A**): *n* = 5; (**B**–**G**): *n* = 15).

Since *d*-limonene was the main component of PE (Figure 1) and was highly correlated to the antifungal activity of PE (Figure 5D), we investigated if *d*-limonene alone was sufficient to suppress *C. gloeosporioides*. We prepared PDA plates incorporated with 1.2–98.8 mM *d*-limonene for in vitro *C. gloeosporioides* antifungal assays. We found that at 24.7 mM or higher concentrations, *d*-limonene completely suppressed *C. gloeosporioides* growth in vitro with an estimated IC_50_ of 10.9 mM (Figure 6A).

We next tested the antifungal effect of *d*-limonene-Tween^®^80 emulsion on anthracnose in mango. In preliminary studies, the effects of 6.2, 12.3, and 24.7 mM *d*-limonene were examined. However, although the fruit treated with Tween^®^80 alone or 6.2 mM *d*-limonene displayed a normal appearance throughout the experiment, treatments of 12.3, and 24.7 mM *d*-limonene caused fruit skin darkening after 2 d (Supplementary Figure S3). Thus, the concentration of *d*-limonene was set at 6.2 mM for subsequent in vivo experiments.

The fruit was immersed in ultrapure water, Tween^®^80, or 6.2 mM *d*-limonene emulsion for 2 min, air dried, and stored at 20 °C for 14 d. Anthracnose symptoms were observed in fruit treated with ultrapure water or Tween^®^80 4 d after storage, while disease symptoms appeared after 8 d of storage in the fruit treated with *d*-limonene (Figure 6B). The disease severity was significantly milder in the fruit treated with Tween^®^80 than in those treated with water after 12 d of storage, whereas the disease severity of the *d*-limonene-treated fruit was significantly milder than those treated with water or Tween^®^80 after 8 or 12 d of storage, respectively (Figure 6B). These findings indicate that *d*-limonene was sufficient to inhibit anthracnose in vivo, while Tween^®^80 might have also contributed to this effect.

The color of the fruit displayed similar changing patterns: the chroma gradually elevated from 42.2 ± 1.9 to 44.9 ± 2.8 (Figure 6C), and the hue angle steadily decreased from 91.1 ± 3.4° to 81.7 ± 3.2° (Figure 6D). The firmness of the fruit dropped from 70.8 ± 18.3 to 3.8 ± 0.8 N (Figure 6E), the total soluble solids increased from 8.9 ± 2.7° to 18.4 ± 1.0° Brix (Figure 6F), and the titratable acids diminished from 0.67 ± 0.04% to 0.17 ± 0.02% (Figure 6G). Taken together, these results demonstrate that Tween^®^80 and *d*-limonene treatments did not alter the ripening physiology of the fruit.

## 3. Discussion

The compositions of pomelo essential oil from different areas have been reported. The major constituents of essential oil isolated from pomelo in Taiwan were 0.8% *α*-pinene, 2.7% *β*-pinene, 3.1% myrcene, 0.1% *α*-phellandrene, 0.4% *β*-ocimene, 87.5% limonene, and 0.4% linalool [17]. The constituents of essential oil isolated from peel of 3 pomelo cultivars in Nepal were 0.2–0.58% *α*-pinene, 0.77–2.17% sabinene, 0.15–6.09% *β*-pinene, 0.74–1.75 myrcene, 63.76–89.15% limonene, 0.35% *β*-phellandrene, 0.13–0.43% *β*-ocimene, and 0.35–2.65% linalool [20]. The constituents of essential oil of pomelo from Tunisia was 0.15% ± 0.06% *α*-pinene, 0.19% ± 0.01% sabinene,1.52% ± 0.06% *β*-pinene, 0.03% ± 0.00% *β*-myrcene, 95.4% ± 3.91% limonene, 0.26% ± 0.09% *β*-ocimene, and 0.09 ± 0.02 [21]. We found the composition of pomelo peel ethanol extract closely resembled that of pomelo peel oils as in the previous reports. This may provide an advantage concerning the agricultural application of pomelo waste since ethanol extraction is relatively simple and requires less equipment and energy input.

The antifungal activities of plant essential oil against mango anthracnose in post-harvest fruit have been reported. *Lippia scaberrima* essential oil applied at concentrations of 0.1–2.4 mL L^−1^ inhibited *C. gloeosporioides* growth in vitro and retarded anthracnose development in ‘Keith’ mango when applied at 2 mL L^−1^ in combination with a wax coating [22]. The authors also investigated the potential active compounds in the essential oil by in vitro assays. It was found that carvone, limonene, and 1,8-cineole (0.1–2.4 mL L^−1^) inhibited the mycelial growth of *C. gloeosporioides* [22]. It was recently reported that essential oil of cinnamon bark, basil, and lemongrass inhibited *C. acutatum* growth in vitro with the minimum inhibitory concentrations of 1.6, 4, and 12 mL L^−1^, respectively. In addition, basil essential oil suppressed anthracnose development in ‘Cat Hoa Loc’ mango inoculated with *C. acutatum*, while cinnamon bark and lemongrass essential oil damaged the fruit skin [23].

We show that PE inhibited *C. gloeosporioides* growth in vitro (IC_50_: 3.2 mL L^−1^) and suppressed anthracnose development in ‘Keitt’ mango (10–20 mL L^−1^). We also found that *d*-limonene inhibited *C. gloeosporioides* in vitro (IC_50_: 10.9 mM) and in vivo (6.2 mM). The required dose for PE was higher than that of *L. scaberrima* and basil essential oil, likely due to the differences in an experimental setting and major constituents. In in vitro fungicidal assays, we cultured *C. gloeosporioides* at 28 °C for 7 d, whereas Regnier et al. [22] conducted the experiment at 23 °C for 6 d. In the in vivo trials, we placed the fruit at 20 °C for 14 d. In contrast, Regnier et al. [22] and Danh et al. [23] sterilized the fruit with 70% ethanol, inoculated the fruit with the pathogens, and observed fungal growth for 6 d and 3–7 d after storage, respectively.

Notably, although we and others showed that limonene inhibited *Colletotrichum* species in vitro [22,24,25,26], little is known about the effect of limonene on suppressing anthracnose in post-harvest crops. It was reported that while limonene had a minor effect on suppressing *C. gloeosporioides* growth in vitro, it significantly reduced the lesion size of anthracnose in immature pepper fruit [27]. Here we provide the evidence demonstrating that PE and *d*-limonene exhibited substantial protective effects against anthracnose in post-harvest mango. However, it was reported that orange peel essential oil (20 mL L^−1^) consisting of 87.2% limonene did not inhibit *C. acutatum* growth in an in vitro assay [23]. The discrepancies might be due to the synergistic effect of different combinations of constituents presented in PE and the orange peel essential oil. Furthermore, we also observed that at over 12.3 mM, *d*-limonene caused pitting and darkening of fruit skin, similar to the fruit skin damage caused by cinnamon and lemongrass essential oil applied at 1.6 and 12 mL L^−1^, respectively [23].

In addition, Huang et al. [28] found that the essential oil of *Artemisia scoparia* inhibited *C. gloeosporioides* growth in vitro with an EC_50_ of 9.32 mL L^−1^. In the in vivo experiments, mango fruit was placed in a container containing 5.3–12.8 μL L^−1^ *A. scoparia* oil, which suppressed anthracnose development by 66.23% and 92.06% in the inoculation and the paroxysm tests, respectively. Chitosan coating incorporated with *Ruta graveolens* essential oil was also reported to inhibit anthracnose development in guava [29] and papaya [30]. A mechanistic study demonstrated that *R. graveolens* coatings upregulated defense-related genes in papaya [31]. Further experiments are required to elucidate the mechanisms involved in the protective effect of PE on mango and the antifungal effect on *C. gloeosporioides*.

Since *d*-limonene is the major constituent in PE and the application of *d*-limonene had similar antifungal activities in vitro and in vivo, *d*-limonene likely acted as the main antifungal compound in PE. Indeed, *d*-limonene has been reported as an antifungal compound against fungal pathogens such as *Candida* species [32,33] and *Austropuccinia psidii* [34], *C. gloeosporioides* [22,27], *Botrytis cinerea* [22,35] and *Penicillium digitatum* [36]. Nonetheless, other constituents such as pinene, myrcene, ocimene, and linalool might also exert the antifungal effect.

Interestingly, it was reported that (+)-*α*-pinene and (+)-*β*-pinene exerted antimicrobial activities against *C. albicans*, *Cryptococcus neoformans*, *Rhizopus oryzae,* and methicillin-resistant *S. aureus* with a minimal inhibitory concentration of 117–4150 mg L^−1^ [37], which is much higher than the concentration found in PE (Table 1). On the other hand, it was reported that of the 33 terpenes commonly found in plant essential oils, only 16 exerted antimicrobial activities. In that report, at a concentration of 0.25 g L^−1^, only R-(+)-limonene and (±)-Linalool showed antimicrobial activity against *B. cereus*, Salmonella spp., *E. coli*, and *S. aureus*, whereas (+)-*α*-pinene, sabinene, (+)-*β*-pinene, *β*-myrcene, or ocimene showed no antimicrobial activity [38]. Taken together, these studies support that *d*-limonene is the key antimicrobial constituent in pomelo.

Despite extensive research conducted to investigate the composition and antimicrobial effects of essential oil, only a handful of studies describe the effect of storage conditions on the composition of essential oil preparations. It was reported that preheating and storage temperature affected the aroma profile of citrus essential oil. The magnitude of alteration in the aroma of citrus oil was mainly determined by the abundance of *d*-limonene, which decreased by 11.0%, 73.4%, and 88.4% after 15 d of storage at 4, 25, and 37 °C, respectively [39]. The stability of *d*-limonene nanoemulsion stored under 4 and 28 °C for 14 d was investigated in a previous report. It was found that the turbidity of the *d*-limonene emulsion increased more drastically and had a slightly larger droplet size when stored at 28 °C, indicating that higher storage temperature resulted in more drastic alterations of the physico-chemical composition of the emulsion. However, the degree of *d*-limonene degradation was not examined [40]. The stability of limonene in lemon-flavored hard tea stored under different temperatures was studied. It was found that limonene concentration decreased by 23–31%, 57–60%, 66–74%, and 77–87% when the samples were stored at ambient 35, 40, and 45 °C, respectively [41].

Here we provide a complete investigation of the changes in composition and antifungal activity of PE samples stored at different temperatures with or without protection from light. In agreement with previous reports, we found that the decrease in the concentration of the main constituents was alleviated by lowering the storage temperature. The preservation of the main constituents led to the higher antifungal activity of the PE samples stored at a lower temperature. On the other hand, we revealed that light conditions had a minor effect on preserving the chemical composition and antifungal activity of PE.

In conclusion, we demonstrated that PE and *d*-limonene inhibited *C. gloeosporioides* in vitro with IC_50_ of 3.2 mL L^−1^ and 10.9 mM, respectively. Treatment of chitosan coating incorporated with 20 mL L^−1^ PE or application of 6.2 mM *d*-limonene emulsion suppressed anthracnose development in ‘Keitt’ mango during storage at 20 °C for 14 d. The treatments significantly delayed the onset and reduced the severity of the disease without causing any adverse effect on the ripening physiology of the fruit. We also showed that storage at temperatures below 5 °C significantly improved the retention of the major constituents and the antifungal activity of the PE samples. These findings shed light on the control of anthracnose in post-harvest crops and broaden the application of pomelo waste as an alternative and more sustainable method for disease management in agriculture.

## 4. Material and Methods

### 4.1. Plant Materials

Pomelo cv. ‘Hegang Wentan’ fruit at commercial maturity was purchased from farms in Hualien, Taiwan, and transported to the laboratory. The fruit was inspected for disease incidence and visual defects. Healthy fruit with a normal appearance was used for pomelo extract preparation as described in Section 4.2. Mango cv. ‘Keitt’ fruit at commercial maturity was harvested from farms in Tainan, Taiwan, and immediately transported to the laboratory. Healthy fruit without any symptom of disease incidence or visual defect was used for the coating treatments and storage experiments as described in Section 4.6.

### 4.2. Preparation and Preservation Tests of Pomelo Extract

The flavedo portion was peeled from the pomelo fruit and mixed with 95% ethanol at a mass ratio of 1:1.58. The mixture was homogenized for 40 s using an MX-V288 blender (Panasonic, Tokyo, Japan). The mixture was macerated for 1 d at room temperature and then pressed through a 200-mesh sieve. The crude extract was vacuum-filtered with 0.45 μm PTFE membranes (Pall Corporation, Port Washington, NY, USA) to obtain a clear pomelo extract (PE). A total of 3.4 kg flavedo was stripped from 23.7 kg of fresh pomelo fruit and extracted with 5.3 kg ethanol. The crude extract was filtered to obtain 4.3 kg clear PE. For preservation condition tests, freshly prepared PE was divided into 90 aliquots, which were stored under 10 different conditions, each including 9 aliquots. To examine the effect of storage temperature, 54 PE aliquots were stored in brown glass vials wrapped with aluminum foil to block light exposure and held at 20°, 10°, 5°, 0°, −20°, and −80 °C. To examine the effect of light exposure, 36 PE aliquots were stored in transparent glass vials and held at 0°, 5°, 10°, and 20 °C. The PE aliquots were stored for 1, 3, and 6 months, and 3 PE aliquots from each storage condition were randomly picked out to determine their chemical composition and antifungal activity 1, 3, and 6 months after storage.

### 4.3. Gas Chromatography-Mass Spectrometry Analysis

Chemical compositions of the PE samples were analyzed using an Agilent 7890A Gas Chromatograph equipped with a DB-5ms Ultra Inert capillary column (30 m/0.25 mm/0.25 µm) coupled with a 5975C mass spectrometer (Agilent Technologies, Santa Clara, CA, USA). PE samples were loaded into auto-sampling vials, and 100 μL L^−1^ ethyl decanoate was added to each sample as an internal control. The vials were installed onto the autosampler for analysis through the following setting: the injection port temperature was controlled at 250 °C, the initial oven temperature was held at 60 °C for 5 min and then ramped to 200 °C at a rate of 5 °C min^−1^. The evaporated sample was carried by helium with a flow rate of 1 mL min^−1^. The samples were ionized by electron impact with 70 eV ionization energy at 200 °C and flowed into the mass spectrometer. Mass spectrum acquisitions were performed in the mass range of 45–500 atomic mass units. The identity of the compounds obtained from the mass spectrum was determined by comparing the retention time and the fragmentation pattern with the National Institute of Standards and Technology (NIST) Tandem and Electron Ionization Spectral Libraries. The retention indices (RI) were calculated according to the equation described by Van den Dool and Kratz [42]. The relative quantity of compounds was calculated based on the signal area of the compound normalized with the internal standard 100 μL L^−1^ ethyl decanoate.

### 4.4. Isolation of Colletotrichum gloeosporioides and In Vitro Fungicidal Assay

The conidia of *C. gloeosporioides* were isolated from infected mango fruit. In brief, the surface of the infected fruit was washed with sterilized water and placed into a biosafety cabinet. The infected area was excised and rinsed in 1% sodium hypochlorite for 30 s and then rinsed twice with ultrapure water for a total of 1 min. The conidia on the surface were collected and inoculated to potato dextran agar (PDA) plates, cultured in a dark, humidified chamber at 28 °C. The resulting conidia were collected for DNA extraction and PCR analysis using *Colletotrichum* and *C. gloeosporioides* specific primers (Supplementary Table S1). The identity of the obtained pathogen was confirmed by sequencing the PCR products.

To determine the antifungal effect of PE and *d*-limonene, 0–80 mL L^−1^ PE, an equivalent amount of ethanol, or 0–100 mM *d*-limonene was premixed with the media and prepared into PDA plates. Conidia were collected from *C. gloeosporioides* cultures and prepared into 10^4^–10^6^ mL^−1^ conidia suspension, and inoculated to the PDA plates at a concentration of 500 conidia per plate. The conidia were cultured for 4 d in a dark, humidified chamber at 28 °C, and the antifungal effects were determined based on the relative growth area normalized with the control. The inhibitory curve was established, and IC_50_ concentrations were calculated.

To compare the antifungal effects of PE stored at different light and temperature settings, 4 mL L^−1^ of PE sample from each storage condition was mixed with the media and prepared into PDA plates, and the antifungal effects were determined as described above.

### 4.5. Preparation of Chitosan Coating Incorporated with Pomelo Extract and d-limonene Emulsion

Chitosan solution (CS) was made by dissolving 5 g chitosan (Tokyo Chemical Industry Co. Ltd., Tokyo, Japan) in 1 L 0.5% acetic acid. To prepare chitosan coating incorporated with PE, 10 or 20 mL PE was mixed with 1 L CS, and the solution was stirred at room temperature for 3 h. To prepare *d*-limonene emulsion, 1 mL polyoxyethylene sorbitan monooleate (Tween^®^80; Nacalai Tesque Inc., Kyoto, Japan) was mixed with 1 mL ultrapure water or *d*-limonene (Alfa Aesar, Ward Hill, MA, USA), the mixture was added dropwise into 1 L ultrapure water with constant stirring and homogenized for 3 h at room temperature.

### 4.6. Coating Treatment and Storage Experiments of Mango

The mango fruit was cleaned carefully with water to remove dust, debris, and latex on the fruit skin. The fruit was then dipped into CS, CS incorporated with PE, vehicle Tween^®^80 emulsion, or *d*-limonene emulsion s for 2 min and placed at room temperature for 30 min. After coating treatment, the fruit was moved into ventilated carton boxes lined with sponge pads and stored at 20 °C and 95% RH. The fruit was weighed and examined for disease symptoms 0, 2, 4, 6, 8, 10,12, and 14 d after storage. After storage, the fruit was subjected to firmness, total soluble solids, and titratable acid measurements. Two storage experiments were performed:

In experiment I, a total of 25 fruits were used. Twenty fruits were randomly selected and divided into 4 groups and treated with ultrapure water, CS, CS incorporated with 10 mL L^−1^ PE, or CS incorporated with 20 mL L^−1^ PE, respectively. The firmness, total soluble solids, and titratable acids of the remaining 5 fruits were measured to determine the pre-storage values. In experiment II, a total of 20 fruits were used. Fifteen fruits were randomly selected, divided into 3 groups, and treated with ultrapure water, vehicle Tween^®^80 emulsion, or *d*-limonene emulsion, respectively. The firmness, total soluble solids, and titratable acid of the remaining 5 fruits were measured before storage.

Both experiments I and II were repeated 3 times on different days, and the data from all 3 repeats were pooled for statistical analysis as described in Section 4.8.

### 4.7. Determination of Disease Index, Firmness, Total Soluble Solids, and Titratable Acids

The disease index (DI) was determined based on the infected area. The DI score of fruit without infection, with an infected area of <10%, 11–20%, 21–35%, or 36–50%, were designated with scores 0, 1, 2, 3, or 4, respectively. The firmness of the fruit was determined by penetration tests using a TA.XTplus Texture Analyser equipped with a flat-end 6 mm probe (Stable Micro Systems, Surrey, UK). Penetration distance and speed were set at 12 mm and 20 mm s^−1^, respectively. The total soluble solids of the pulps were determined by a PAL-1 digital refractometer (ATAGO Co., Ltd., Tokyo, Japan). To determine the titratable acids, 5 g of pulps was diluted with 75 g ultrapure water, and automated titration was conducted using a Titrando 905 equipped with Robotic Titrosampler (Metrohem, Herisau, Switzerland). Sodium hydroxide was used as the titrant, and the titration was terminated at a pH of 8.4. The volumes of titrant used were recorded and calculated to citric acid equivalents.

### 4.8. Statistical Analysis

All experiments were repeated independently at least 3 times. The data from 3 independent experiments were pooled for statistical analysis and presented as mean ± standard deviation or standard error of the mean. The numbers of biological replicates in each figure panel are indicated in the legends. A significant difference (*p* < 0.05) between data was determined by Tukey’s tests using Prism 8 software (GraphPad Software, San Diego, CA, USA).

## Figures and Tables

**Table 1:** The retention time (RT), abundance, concentration, and retention indices (RI) of the major constituents in pomelo extracts identified by GC-MS analysis.

Peak	Compound	RT	Abundance	Conc.	RI ^a^
		(min)	(% total)	(μL L^−1^)	
1	*α*-pinene	8.54	0.37	36.0	931
2	Sabinene	9.91	0.37	36.3	973
3	*β*-pinene	10.03	1.79	175.3	977
4	*β*-myrcene	10.48	0.91	89.2	991
5	*d*-limonene	11.88	76.11	7445.9	1032
6	*β*-ocimene	12.45	0.51	49.5	1049
7	Linalool	14.17	0.31	30.8	1101
8	DDMP *	15.58	0.78	76.5	1146

* 2,3-Dihydro-3,5-dihydroxy-6-methyl-4(H)-pyran-4-one; ^a^ C7–C30 saturated alkanes standards were used as references to calculate RI.

## Data Availability

Not applicable.

## Supplementary Materials

The following supporting information can be downloaded at: https://www.mdpi.com/article/10.3390/plants11152064/s1, Figure S1: The inhibition curve and IC_50_ of ethanol against *C. gloeosporioides* determined by in vitro assay; Figure S2: Ethanol damages the fruit skin of ‘Keitt’ mango; Figure S3: High concentration of *d*-limonene damages the fruit skin of ‘Keitt’ mango; Table S1: Primers for *Colletotrichum* spp. and *C. gloeosporioides*.

## References

[1] Xiao L., Ye F., Zhou Y., Zhao G. (2021). Utilization of Pomelo Peels to Manufacture Value-Added Products: A Review. Food Chem..

[2] Tocmo R., Pena-Fronteras J., Calumba K.F., Mendoza M., Johnson J.J. (2020). Valorization of Pomelo (*Citrus grandis* Osbeck) Peel: A Review of Current Utilization, Phytochemistry, Bioactivities, and Mechanisms of Action. Compr. Rev. Food Sci. Food Saf..

[3] Sivakumar D., Bautista-Baños S. (2014). A Review on the Use of Essential Oils for Postharvest Decay Control and Maintenance of Fruit Quality during Storage. Crop Prot..

[4] Lima N.B., Batista M.V.d.A., De Morais M.A., Barbosa M.A.G., Michereff S.J., Hyde K.D., Câmara M.P.S. (2013). Five *Colletotrichum* Species are Responsible for Mango Anthracnose in Northeastern Brazil. Fungal Divers..

[5] Swart S.H., Serfontein J.J., Swart G., Labuschagne C. (2009). Chemical Control of Post-harvest Diseases of Mango: The Effect of Fludioxonil and Prochloraz on Soft Brown Rot, Stem-End Rot and Anthracnose. Acta Hortic..

[6] Henriod R., Diczbalis Y., Sole D., Stice K.N., Tora L. (2016). Investigation into Various Fungicides and Alternative Solutions for Controlling Postharvest Diseases in Papaya Fruit. Acta Hortic..

[7] Shimshoni J.A., Bommuraj V., Chen Y., Sperling R., Barel S., Feygenberg O., Maurer D., Alkan N. (2020). Postharvest Fungicide for Avocado Fruits: Antifungal Efficacy and Peel to Pulp Distribution Kinetics. Foods.

[8] De la Rosa-García S.C., Martínez-Torres P., Gómez-Cornelio S., Corral-Aguado M.A., Quintana P., Gómez-Ortíz M. (2018). Antifungal activity of ZnO and MgO nanomaterials and their mixtures against *Colletotrichum gloeosporioides* strains from Tropical Fruit. J. Nanomater..

[9] Meena M., Pilania S., Pal A., Mandhania S., Bhushan B., Kumar S., Gohari G., Saharan V. (2020). Cu-chitosan Nano-net Improves Keeping Quality of Tomato by Modulating Physio-Biochemical Responses. Sci. Rep..

[10] Marques K.M., Galati V.C., Fernandes J.D.R., Guimarães J.E.R., Silva J.P., Mattiuz B.H., Mattiuz C.F.M. (2016). Use of Chitosan for the Control of Postharvest Anthracnose and Quality in Avocados. Acta Hortic..

[11] Sarkhosh A., Schaffer B., Vargas A.I., Palmateer A.J., Lopez P., Soleymani A., Farzaneh M. (2018). Antifungal Activity of Five Plant-Extracted Essential Oils against Anthracnose in Papaya Fruit. Biol. Agric. Hortic..

[12] Dias B.L., Costa P.F., Dakin M.S., de Souza Carlos Mou rao D., Dias F.R., de Sousa R.R., Ped ro Raym undo Argiielles O., Talita Pereira de Souza F., Fabricio S.C., Gil Rod rigues Dos S. (2020). Control of Papaya Fruits Anthracnose by Essential Oils of Medicinal Plants Associated to Different Coatings. J. Med. Plants Res..

[13] Ali A., Wee Pheng T., Mustafa M.A. (2015). Application of Lemongrass Oil in Vapour Phase for the Effective Control of Anthracnose of ‘Sekaki’ Papaya. J. Appl. Microbiol..

[14] Chillet M., Minier J., Ducrog M., Meile J.C. (2018). Postharvest Treatment of Mango: Potential Use of Essential Oil with Thymol to Control Anthracnose Development. Fruits.

[15] Esquivel-Chávez F., Colín-Chávez C., Virgen-Ortiz J.J., Martínez-Téllez M.A., Avena-Bustillos R.J., Peña-Madrigal G., Miranda-Ackermanf M.A. (2021). Control of Mango Decay Using Antifungal Sachets Containing of Thyme Oil/Modified Starch/Agave Fructan Microcapsules. Future Foods.

[16] Correa-Pacheco Z.N., Bautista-Baños S., Valle-Marquina M.Á., Hernández-López M. (2017). The Effect of Nanostructured Chitosan and Chitosan-Thyme Essential Oil Coatings on *Colletotrichum gloeosporioides* Growth in vitro and on cv Hass avocado and Fruit Quality. J. Phytopathol..

[17] Chen G.-W., Lin Y.-H., Lin C.-H., Jen H.-C. (2018). Antibacterial Activity of Emulsified Pomelo (*Citrus grandis* Osbeck) Peel Oil and Water-Soluble Chitosan on *Staphylococcus aureus* and *Escherichia coli*. Molecules.

[18] Deng W., Liu K., Cao S., Sun J., Zhong B., Chun J. (2020). Chemical Composition, Antimicrobial, Antioxidant, and Antiproliferative Properties of Grapefruit Essential Oil Prepared by Molecular Distillation. Molecules.

[19] Abdel-Aziz M.M., Emam T.M., Elsherbiny E.A. (2019). Effects of Mandarin (*Citrus reticulata*) Peel Essential Oil as a Natural Antibiofilm Agent against *Aspergillus niger* in Onion Bulbs. Postharvest Biol. Technol..

[20] Bhandari D.P., Poudel D.K., Satyal P., Khadayat K., Dhami S., Aryal D., Chaudhary P., Ghimire A., Parajuli N. (2021). Volatile Compounds and Antioxidant and Antimicrobial Activities of Selected Citrus Essential Oils Originated from Nepal. Molecules.

[21] Hosni K., Zahed N., Chrif R., Abid I., Medfei W., Kallel M., Brahim N.B., Sebei H. (2010). Composition of Peel Essential Oils from Four Selected Tunisian Citrus Species: Evidence for the Genotypic Influence. Food Chem..

[22] Regnier T., du Plooy W., Combrinck S., Botha B. (2008). Fungitoxicity of *Lippia scaberrima* essential oil and selected terpenoid components on two mango postharvest spoilage pathogens. Postharvest Biol. Technol..

[23] Danh L.T., Giao B.T., Duong C.T., Nga N.T.T., Tien D.T.K., Tuan N.T., Huong B.T.C., Nhan T.C., Trang D.T.X. (2021). Use of Essential Oils for the Control of Anthracnose Disease Caused by *Colletotrichum acutatum* on Post-Harvest Mangoes of Cat Hoa Loc Variety. Membranes.

[24] Quintana-Rodriguez E., Rivera-Macias L.E., Adame-Alvarez R.M., Torres J.M., Heil M. (2018). Shared Weapons in Fungus-fungus and Fungus-plant Interactions? Volatile Organic Compounds of Plant or Fungal Origin Exert Direct Antifungal Activity in vitro. Fungal Ecol..

[25] Feng J., Wang R., Chen Z., Zhang S., Yuan S., Cao H., Jafari S.M., Yang W. (2020). Formulation Optimization of *D*-limonene-loaded Nanoemulsions as a Natural and Efficient Biopesticide. Colloids Surf. A Physicochem. Eng. Asp..

[26] Scariot F.J., Foresti L., Delamare A.P.L., Echeverrigaray S. (2020). Activity of Monoterpenoids on the in vitro Growth of two *Colletotrichum* species and the Mode of Action on *C. acutatum*. Pestic. Biochem. Physiol..

[27] Hong J.K., Yang H.J., Jung H., Yoon D.J., Sang M.K., Jeun Y.C. (2015). Application of Volatile Antifungal Plant Essential Oils for Controlling Pepper Fruit Anthracnose by *Colletotrichum gloeosporioides*. Plant Pathol. J..

[28] Huang X., Liu T., Zhou C., Huang Y., Liu X., Yuan H. (2021). Antifungal Activity of Essential Oils from Three *Artemisia* Species Against *Colletotrichum gloeosporioides* of Mango. Antibiotics.

[29] Grande Tovar C.D., Delgado-Ospina J., Navia Porras D.P., Peralta-Ruiz Y., Cordero A.P., Castro J.I., Chaur Valencia M.N., Mina J.H., Chaves López C. (2019). *Colletotrichum gloesporioides* Inhibition in situ by Chitosan-*Ruta graveolens* Essential Oil Coatings: Effect on Microbiological, Physicochemical, and Organoleptic Properties of Guava (*Psidium guajava* L.) during Room Temperature Storage. Biomolecules.

[30] Peralta-Ruizm Y., Grande Tovar C., Sinning-Mangonez A., Bermont D., Pérez Cordero A., Paparella A., Chaves-López C. (2020). Colletotrichum gloesporioides Inhibition Using Chitosan-Ruta graveolens L Essential Oil Coatings: Studies In Vitro and In Situ on Carica papaya Fruit. Int. J. Food Microbiol..

[31] Landi L., Peralta-Ruiz Y., Chaves-López C., Romanazzi G. (2021). Chitosan Coating Enriched with *Ruta graveolens* L. Essential Oil Reduces Postharvest Anthracnose of Papaya (*Carica papaya* L.) and Modulates Defense-Related Gene Expression. Front. Plant Sci..

[32] Muñoz J.E., Rossi D.C.P., Jabes D.L., Barbosa D.A., Cunha F.F.M., Nunes L.R., Arruda D.C., Pelleschi Taborda C. (2020). In Vitro and In Vivo Inhibitory Activity of Limonene against Different Isolates of *Candida* spp.. J. Fungi.

[33] Yu H., Lin Z.-X., Xiang W.-L., Huang M., Tang J., Lu Y., Zhao Q.-H., Zhang Q., Rao Y., Liu L. (2022). Antifungal Activity and Mechanism of *D*-Limonene against Foodborne Opportunistic Pathogen *Candida tropicalis*. LWT Food Sci. Technol..

[34] Silva R.R., da Silva A.C., Rodella R.A., Marques M.O.M., Zanuncio A.J.V., Soares M.A., Serrão J.E., Zanuncio J.C., Furtado E.L. (2020). Limonene, a Chemical Compound Related to the Resistance of Eucalyptus Species to *Austropuccinia psidii*. Plant Dis..

[35] Umagiliyage A.L., Becerra-Mora N., Kohli P., Fisher D.J., Choudhary R. (2017). Antimicrobial Efficacy of Liposomes Containing *D*-Limonene and its Effect on the Storage Life of Blueberries. Postharvest Biol. Technol..

[36] Du Plooy W., Regnier T., Combrinck S. (2009). Essential Oil Amended Coatings as Alternatives to Synthetic Fungicides in Citrus Postharvest Management. Postharvest Biol. Technol..

[37] Da Silva A.C.R., Lopes P.M., Azevedo M.M.B.D., Costa D.C.M., Alviano C.S., Alviano D.S. (2012). Biological Activities of *α*-Pinene and *β*-Pinene Enantiomers. Molecules.

[38] Guimarães A.C., Meireles L.M., Lemos M.F., Guimarães M.C.C., Endringer D.C., Fronza M., Scherer R. (2019). Antibacterial Activity of Terpenes and Terpenoids Present in Essential Oils. Molecules.

[39] Yang Y., Zhao C., Tian G., Lu C., Zhao S., Bao Y., McClements D.J., Xiao H., Zheng J. (2017). Effects of Preheating and Storage Temperatures on Aroma Profile and Physical Properties of Citrus-Oil Emulsions. J. Agric. Food Chem..

[40] Zahi M.R., Wan P., Liang H., Yuan Q. (2014). Formation and Stability of *D*-Limonene Organogel-Based Nanoemulsion Prepared by a High-Pressure Homogenizer. J. Agric. Food Chem..

[41] He F., Qian Y.L., Qian M.C. (2018). Flavor and Chiral Stability of Lemon-Flavored Hard Tea during Storage. Food Chem..

[42] Van den Dool H., Kratz P.D. (1963). A Generalization of the Retention Index System Including Linear Temperature Programmed Gas-Liquid Partition Chromatography. J. Chromatogr. A.

