# Supplementary material for: Impact of Storage Condition on Chemical Composition and Antifungal Activity of Pomelo Extract against Colletotrichum gloeosporioides and Anthracnose in Post-harvest Mango
Source: Plants (Basel). 2022 Aug 7;11(15):2064. doi: 10.3390/plants11152064 (PMC9370353; doi:10.3390/plants11152064)
Supplement: Supplementary file 1 [file plants-11-02064-s001.zip › plants-1810315-supplementary.pdf]

Supplementary Material

Supplementary Table S1. Primers for *Colletotrichum* spp. and *C. gloeosporioides*.

Species	Primer	Sequence	Amplicon
Colletotrichum	Coll-F	CTATAACTGTTGCTTCGGCG	447 bp
	Coll-R	AAATTTGGGGGTTTTACGGC	
C. gloeosporioides	CgsF1	GGCGGGTAGGGTCTCCGTGAC	310 bp
	CgsR1	TTTGAGGGCCTACATCAGCT	

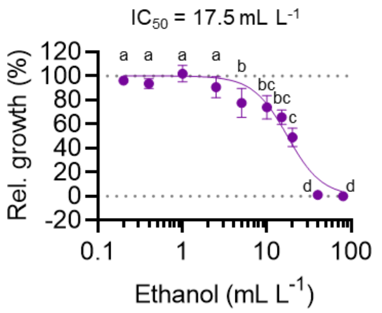

Supplementary Figure S1. The inhibition curve and IC_{50} of ethanol against *C. gloeosporioides* determined by *in vitro* assay. Data is presented as mean ± SD, and the significant differences are indicated by different alphabets (Tukey's tests, $p < 0.05$; $n = 5$).

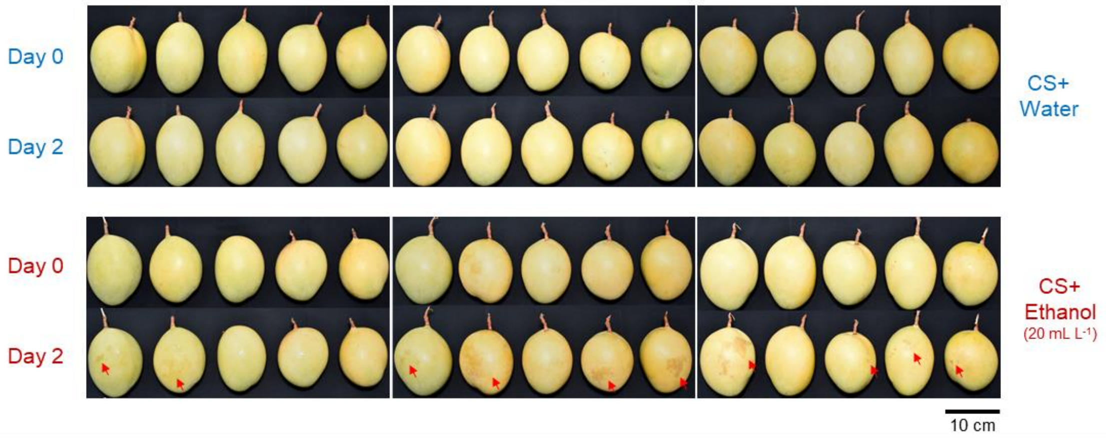

Supplementary Figure S2. Ethanol damages the fruit skin of 'Keitt' mango. Mango fruit were treated with chitosan (CS; top panel) or CS fused with 20 mL L^{-1} ethanol (bottom panel). Pitting and fruit skin darkening appeared 2 d after storage and are indicated by red arrows.

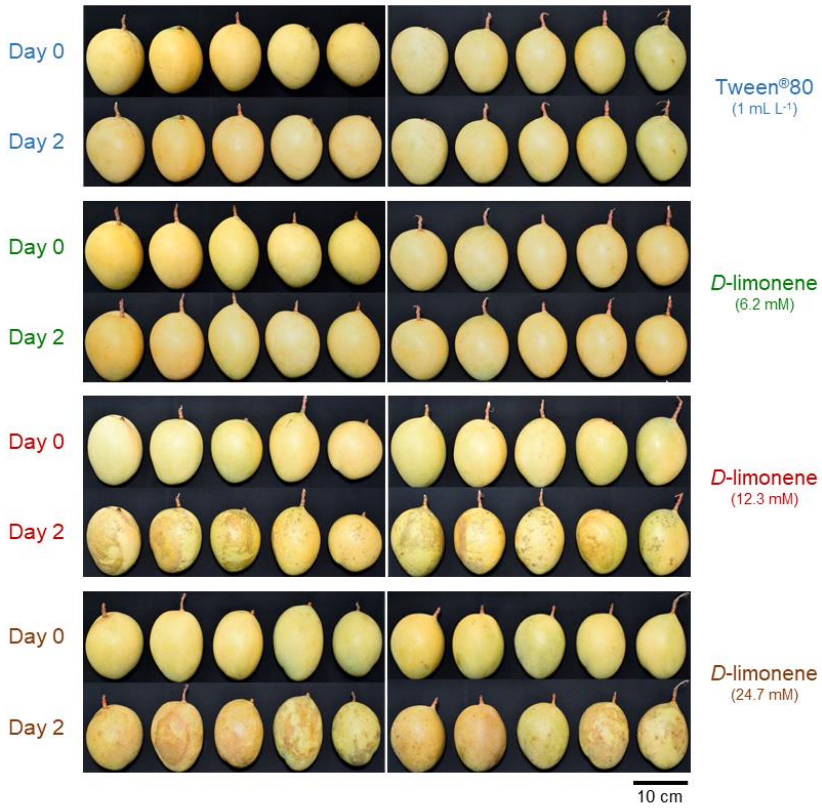

Supplementary Figure S3. High concentration of *d*-limonene damages the fruit skin of 'Keitt' mango. Mango fruit were treated with emulsions of 1 mL L^{-1} Tween®80 or 1 mL L^{-1} Tween®80 fused with 6.2, 12.3, or 24.7 mM *d*-limonene. Severe damage of fruit skin appeared 2 d after storage in fruit treated with 12.3 or 24.7 mM *d*-limonene emulsion.
